# Special Issue on Clinical Medicine for Healthcare and Sustainability

**DOI:** 10.3390/jcm9072206

**Published:** 2020-07-13

**Authors:** Teen-Hang Meen, Yusuke Matsumoto, Kuan-Han Lee

**Affiliations:** 1 Department of Electronic Engineering, National Formosa University, Yunlin 632, Taiwan; 2 Department of Pharmacy, Tokyo University of Pharmacy and Life Sciences, 1432-1 Horinouchi, Hachioji-city, Tokyo 192-0392, Japan; y-matsu@toyaku.ac.jp; 3 Department of Pharmacy, Chia Nan University of Pharmacy & Science, Tainan City 717, Taiwan; kuanhanlee@mail.cnu.edu.tw

**Keywords:** healthcare and sustainability, therapy of internal medicine diseases, cardiometabolic diseases

## Abstract

Recently, due to the advancement of network technology, big data and artificial intelligence, the healthcare industry has undergone many sector-wide changes. Medical care has not only changed from passive and hospital-centric to preventative and personalized, but also from disease-centric to health-centric. Healthcare systems and basic medical research are becoming more intelligent and being implemented in biomedical engineering. This Special Issue on “Clinical Medicine for Healthcare and Sustainability” selected 30 excellent papers from 160 papers presented in IEEE ECBIOS 2019 on the topic of clinical medicine for healthcare and sustainability. Our purpose is to encourage scientists to propose their experiments and theoretical researches to facilitate the scientific prediction and influential assessment of global change and development.

## 1. Introduction

Due to the development of technology and the advancements in medicine and healthcare, the average life expectancy of human beings has been on the rise for a long time. However, both the fertility rate and the mortality rate have fallen, resulting in the overall population structure rapidly aging, and it has been officially entered an advanced age society. Moreover, as the family’s care function gradually fades, the pressure of personal and family care is increasing, which in turn leads to social and economic problems. Therefore, establishing a perfect long-term system of healthcare and sustainability has become one of the key factors to a complete social security system.

Therefore, the 2019 IEEE Eurasian Biomedical Engineering, Healthcare and Sustainability Conference (IEEE ECBIOS 2019) was held in Okinawa, Japan from 31 May to 3 June 2019, providing researchers in the field of biomedical engineering with a unified communication platform for healthcare and sustainability. Recently, due to the developments of computing, network technology, big data, and artificial intelligence, the healthcare industry has undergone a cross-industry transformation. Medical care has not only changed from response-centric and hospital-centric to preventative and personalized, but also from disease-centric to health-centric. Healthcare systems and basic medical research are becoming more intelligent and being implemented in biomedical engineering. This special issue of “Health Care and Sustainable Clinical Medicine” selected 30 excellent papers from 160 papers published in IEEE ECBIOS 2019, with the theme of healthcare and sustainable clinical medicine. It connects multiple disciplines, including clinical laboratory diagnosis and the treatment of medical diseases, traumatology and precision surgical techniques, clinical cancer research, neurology and psychiatry, dermatology, medical imaging, nuclear medicine, genomics, proteomics and bioinformatics, as well as medicine and women’s health. Our aim is to encourage scientists to publish their experiments and theoretical studies to promote scientific predictions and impact assessments of global change and development.

## 2. The Topics of Clinical Medicine for Healthcare and Sustainability

This Special Issue on “Clinical Medicine for Healthcare and Sustainability” selected 30 excellent papers from 160 papers presented in IEEE ECBIOS 2019 on the topic of clinical medicine for healthcare and sustainability. The topics of published papers are listed in Table 1.

## 3. Conclusions

When the domestic government, the private sector, and people in various professional fields talk about related long-term care issues, they all focus on creating a warm and home-like care institution. However, we actively emphasize the importance of community-based long-term care. While implementing the goal of “aging in place”, the development of domestic non-institutional care is still in its infancy, and the satisfaction of some long-term care needs must still be completed through institutional care, and the extension or outreach of community-based care, as well as a respite service platform for the development of community-based long-term care, still rely on institutional care to help facilitate it. The development of long-term care in Taiwan is much shorter than that of Japan, Europe, the United States, and Canada. Despite years of hard work and rapid development, the long-term care resources needed to establish a complete system in terms of universalization, fairness, accessibility, and selectivity are not available. It is hoped that in the future, based on the soundness of institutional care, the outreach will move towards the goals of “community care” and “aging in place”. We hope the researches of this special issue can improve the developments of clinical medicine for healthcare and sustainability.

## Figures and Tables

**Table 1:** The topics and list of papers for the Special Issue on “Clinical Medicine for Healthcare and Sustainability”.

Topics	Papers of Special Issue
Clinical Laboratory Diagnosis and Therapy of Internal Medicine Diseases	Kuwabara et al. [1], Daniel et al. [2], Lee et al. [3], Ye et al. [4], Jang [5], Lee et al. [6], Jiang et al. [7], Kapur et al. [8], Park et al. [9], Encarnación et al. [10], Wang et al. [11], Lan et al. [12], Chun et al. [13], Chen et al. [14], Jurik et al. [15], Lai et al. [16], Lin et al. [17], Caneiras et al. [18]
Traumatology and Precise Surgical Techniques	Chiu et al. [19]
Genomics, Proteomics, and Bioinformatics in Clinical Cancer Research	Kong et al. [20], Chiu et al. [21]
Neurological and Psychiatric Disorders	Kume et al. [22], Huh et al. [23], Ricardo et al. [24]
Advanced Research in Dermatology	Damiani et al. [25]
Medical Imaging and Nuclear Medicine	Lee et al. [26], Shiao et al. [27], Jo et al. [28]
Rehabilitation Medicine	Yang et al. [29]
Women’s Health	Lee et al. [30]

## References

[1] Kuwabara M., Hisatome I., Niwa K., Bjornstad P., Carlos A.R.J., Ana A.H., Kanbay M., Richard J.J., Lanaspa M.A. (2020). The optimal range of serum uric acid for cardiometabolic diseases: A 5-year japanese cohort study. J. Clin. Med..

[2] Daniel P.F., Concepción B.R.L., Concepción P.C.C., Cesar H.M., María P.G.C., Rafael M.J. (2020). Prospective evaluation of intensity of symptoms, therapeutic procedures and treatment in palliative care patients in nursing homes. J. Clin. Med..

[3] Lee S.H., Lim C.M., Koh Y., Hong S.B., Huh J.W. (2020). Effect of an electronic medical record-based screening system on a rapid response system: 8-years’ experience of a single center cohort. J. Clin. Med..

[4] Ye S., Zhang H., Shi F., Guo J., Wang S., Zhang B. (2020). Ensemble learning to Improve the prediction of fetal macrosomia and large-for-gestational Age. J. Clin. Med..

[5] Jang A. (2020). Postprandial hypotension as a risk factor for the development of new cardiovascular disease: A prospective cohort study with 36 month follow-up in community-dwelling elderly people. J. Clin. Med..

[6] Lee M., Park S., Lee K.S. (2020). Relationship between morbidity and health behavior in chronic diseases. J. Clin. Med..

[7] Jiang F.C., Shih C.M., Wang Y.M., Yang C.T., Chiang Y.J., Lee C.H. (2019). Decision support for the optimization of provider staffing for hospital emergency departments with a queue-based approach. J. Clin. Med..

[8] Kapur S., Gehani M., Kammili N., Bhardwaj P., Nag V., Devara S.M., Sharad S. (2019). Clinical validation of innovative optical-sensor-based, low-cost, rapid diagnostic test to reduce antimicrobial resistance. J. Clin. Med..

[9] Park K., Nemoto K., Yamakawa Y., Yamashita F., Yoshida K., Tamura M., Kawaguchi A., Arai T., Sasaki M. (2019). cerebral white matter hyperintensity as a healthcare quotient. J. Clin. Med..

[10] Encarnación B.R., Valdellós J., Ricardo O.R., María R.G.M., Lorena A.C., Gabriel A.Z., Inmaculada B.E. (2019). factors associated with health-related quality of life in community-dwelling older adults: A multinomial logistic analysis. J. Clin. Med..

[11] Wang C.Y., Hsien H.H., Hung K.W., Lin H.F., Chiou H.Y., Yeh S.C.J., Yeh Y.J., Shi H.Y. (2019). Multidiscipline stroke post-acute care transfer system: Propensity-score-based comparison of functional status. J. Clin. Med..

[12] Lan S.H., Chang S.P., Lai C.C., Lu L.C., Chao C.M. (2019). The efficacy and safety of eravacycline in the treatment of complicated intra-abdominal infections: A systemic review and meta-analysis of randomized controlled trials. J. Clin. Med..

[13] Chun D.I., Kim S., Kim J., Yang H.J., Kim J.H., Cho J.H., Yi Y., Kim W.J., Won S.H. (2019). Epidemiology and burden of diabetic foot ulcer and peripheral arterial disease in korea. J. Clin. Med..

[14] Chen C.H., Chen Y.M., Yang Y., Chang Y.J., Lin L.J., Yen H.C. (2019). Re-evaluating the protective effect of hemodialysis catheter locking solutions in hemodialysis patients. J. Clin. Med..

[15] Jurik R., Stastny P. (2019). Role of nutrition and exercise programs in reducing blood pressure: A systematic review. J. Clin. Med..

[16] Lai C.C., Cheng I.L., Chen Y.H., Tang H.J. (2019). The efficacy and safety of doripenem in the treatment of acute bacterial infections—A systemic review and meta-analysis of randomized controlled trials. J. Clin. Med..

[17] Lin H.T., Li Y.I., Hu W.P., Huang C.C., Du Y.C. (2019). A scoping review of the efficacy of virtual reality and exergaming on patients of musculoskeletal system disorder. J. Clin. Med..

[18] Caneiras C., Jácome C., Sagrario M.A., Calvo J.R., Fonseca J.A., Escarrabill J., Winck J.C. (2019). Patient experience in home respiratory therapies: Where we are and where to go. J. Clin. Med..

[19] Chiu C.C., Lin W.L., Shi H.Y., Huang C.C., Chen J.J., Su S.B., Lai C.C., Chao C.M., Tsao C.J., Chen S.H. (2019). Comparison of oncologic outcomes in laparoscopic versus open surgery for non-metastatic colorectal cancer: Personal experience in a single institution. J. Clin. Med..

[20] Kong S., Park H.Y., Kang D., Lee J.K., Lee G., Kwon O.J., Shim Y.M., Zo J.I., Cho J. (2020). Seasonal variation in physical activity among preoperative patients with lung cancer determined using a wearable device. J. Clin. Med..

[21] Chiu C.C., Lee K.T., Wang J.J., Sun D.P., Lee H.H., Huang C.C., Shi H.Y. (2019). Preoperative health-related quality of life predicts minimal clinically important difference and survival after surgical resection of hepatocellular carcinoma. J. Clin. Med..

[22] Kume Y., Takahashi T., Itakura Y., Lee S., Makizako H., Ono T., Shimada H., Ota H. (2019). Characteristics of mild cognitive impairment in northern japanese community-dwellers from the orange registry. J. Clin. Med..

[23] Huh Y., Nam G.E., Kim Y.H., Lee J.H. (2019). Relationships between multimorbidity and suicidal thoughts and plans among korean adults. J. Clin. Med..

[24] Ricardo J.D.O., Teresa M. (2020). Abuse of licit and illicit psychoactive substances in the workplace: Medical, toxicological, and forensic aspects. J. Clin. Med..

[25] Damiani G., Pacifico A., Russo F., Pigatto P.D.M., Bragazzi N.L., Bonifati C., Morrone A., Watad A., Adawi M. (2019). Use of secukinumab in a cohort of erythrodermic psoriatic patients: A pilot study. J. Clin. Med..

[26] Lee K.S., Jung S.K., Ryu J.J., Shin S.W., Choi J. (2020). Evaluation of transfer learning with deep convolutional neural networks for screening osteoporosis in dental panoramic radiographs. J. Clin. Med..

[27] Shiao E.C., Kuo P.L. (2019). Two-dimensional laser-align device for ultrasound-guided injection. J. Clin. Med..

[28] Jo M.H., Lim T.S., Jeon M.Y., Lee H.W., Kim B.K., Park J.Y., Kim D.Y., Ahn S.H., Han K.H., Kim S.U. (2019). Predictors of discordance in the assessment of skeletal muscle mass between computed tomography and bioimpedance analysis. J. Clin. Med..

[29] Yang C., Mustafa R., Scott M., Johan O., Ingmar N. (2019). Predicting long-term health-related quality of life after bariatric surgery using a conventional neural network: A study based on the scandinavian obesity surgery registry. J. Clin. Med..

[30] Lee J.H., Go T.H., Lee S.H., Kim J., Huh J.H., Kim J.Y., Kang D.R., Jeong S., Koh S.B., Choi J.R. (2019). Association between serum urate and risk of hypertension in menopausal women with xdh gene. J. Clin. Med..

